# Submucosal tunneling endoscopic septum division for Zenker’s diverticulum in a patient with scoliosis and stent impaction

**DOI:** 10.1055/a-2743-9321

**Published:** 2025-12-08

**Authors:** Zhi-Yu Xiang, Lina Fan, Sheng-Li Lin, Lili Ma, Ping-Hong Zhou

**Affiliations:** 1 92323 Endoscopy Center and Endoscopy Research Institute, Zhongshan Hospital, Fudan University, Shanghai, China; 2 92323 Nursing Department, Zhongshan Hospital, Fudan University, Shanghai, China; 3 Shanghai Collaborative Innovation Center of Endoscopy, Shanghai, China

A 72-year-old woman was admitted because of dysphagia and recurrent chest pain for the past 6 months. Two years ago, she was diagnosed with Zenker’s diverticulum at a local hospital and underwent esophageal stent placement for occlusion. The patient also presented with severe congenital scoliosis ( Fig. 1 **a, b** ), with a height of 115 cm and a weight of 35 kg. Despite the initial intervention, her symptoms persisted with worsening dysphagia, chest pain, and multiple episodes of aspiration pneumonia over the past 6 months. Endoscopic evaluation revealed stent migration and impaction, with unsuccessful removal attempts at local hospital ( Fig. 1 **c** ). The endoscopic stent retrieval combined with submucosal tunneling endoscopic septum division (STESD) was proposed after a multidisciplinary discussion with the anesthesia, orthopedic, and respiratory departments.

**Fig. 1:**
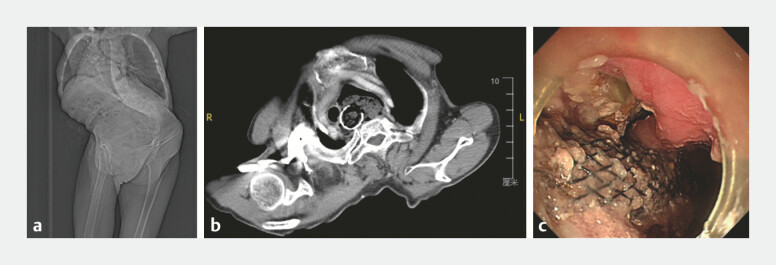
Images showing significantly increased challenges and risks. **a, b** Radiographic images showing severe congenital scoliosis. **c** The displaced and impacted stent filled with food.

The procedure involved five steps ( Video 1 ). First, granulation was dissected with a IT knife at the impacted stent site and stent retrieval was successfully achieved ( Fig. 2 **a** ). Second, a mucosal incision was created 3 cm proximal to the diverticular septum ( Fig. 2 **b** ). Third, the submucosal longitudinal tunneling was established using a hybrid knife ( Fig. 2 **c, d** ). As the scoliosis caused esophageal tortuosity, the tunnel was created according to the direction of the esophageal travel. Fourth, complete transection of the diverticular septum was performed ( Fig. 2 **e** ). Fifth, the mucosal entry was closed with endoclips after hemostasis ( Fig. 2 **f** ).

Submucosal tunneling endoscopic septum division for Zenker’s diverticulum in a patient with scoliosis and stent impaction.Video 1

**Fig. 2:**
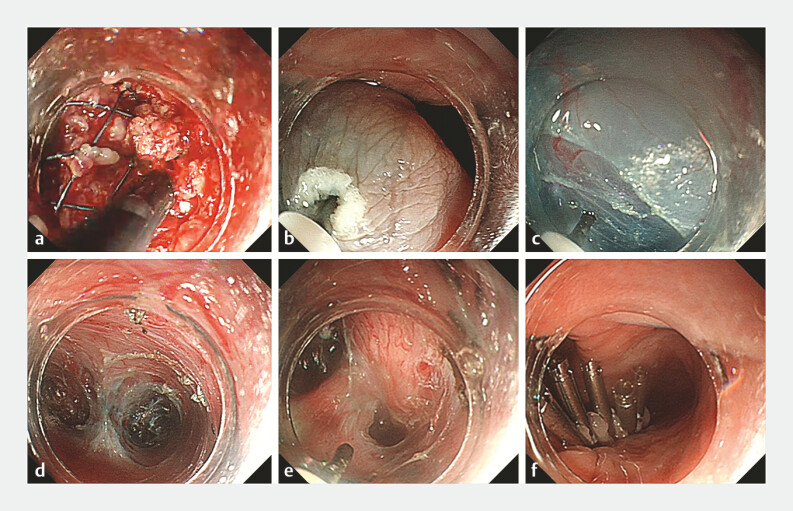
Endoscopic images showing **a** separation of the granulation tissue around the esophageal stent. **b** A mucosal incision. **c** The submucosal longitudinal tunneling. **d** Tunneling performed at both sides of the septum. **e** The septum being prepared for complete transection. **f** The mucosal entry closed with endoclips after hemostasis.

The procedure duration was 80 minutes. The patient was discharged on postoperative day 8 after an uneventful recovery. One-month postoperative esophagography demonstrated restored luminal patency without contrast retention ( Fig. 3 ).

**Fig. 3:**
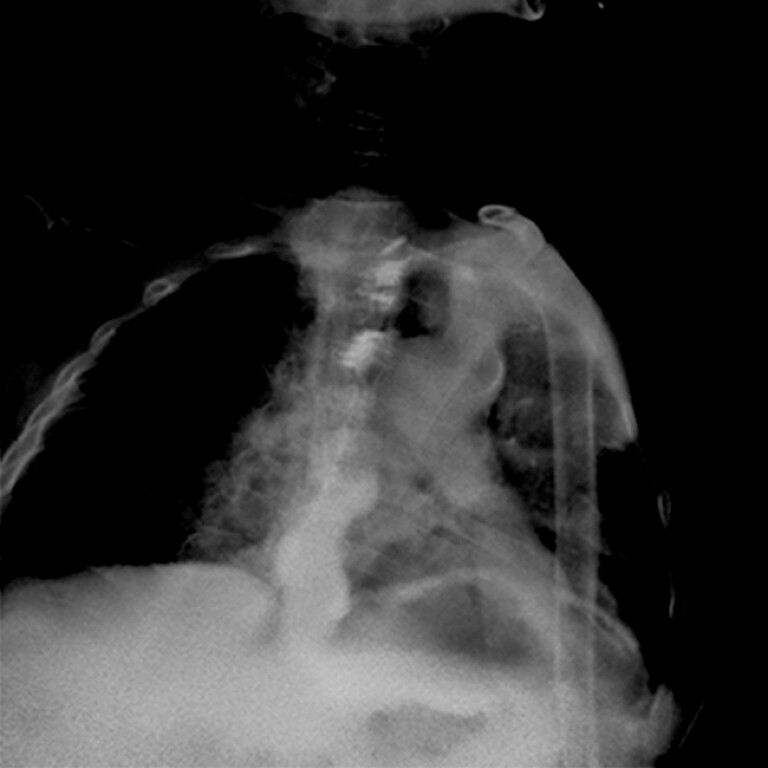
Esophagography showing restored luminal patency without contrast retention.

STESD has become widely accepted as a minimally invasive procedure for treating Zenker’s diverticulum 1 . We herein report the first case of Zenker’s diverticulum in a patient with severe scoliosis complicated by stent migration and impaction managed by STESD. The procedure demonstrated significantly increased risks. This case highlights the importance of tailored minimally invasive technique selection and multidisciplinary collaboration.

Endoscopy_UCTN_Code_TTT_1AO_2AL

## Notes

Endoscopy E-Videos https://eref.thieme.de/e-videos
*E-Videos* is an open access online section of the journal *Endoscopy* , reporting on interesting cases and new techniques in gastroenterological endoscopy. All papers include a high-quality video and are published with a Creative Commons CC-BY license. Endoscopy E-Videos qualify for HINARI discounts and waivers and eligibility is automatically checked during the submission process. We grant 100% waivers to articles whose corresponding authors are based in Group A countries and 50% waivers to those who are based in Group B countries as classified by Research4Life (see: https://www.research4life.org/access/eligibility/ ). This section has its own submission website at https://mc.manuscriptcentral.com/e-videos .
